# Big Lessons from Tiny Flies: *Drosophila melanogaster* as a Model to Explore Dysfunction of Dopaminergic and Serotonergic Neurotransmitter Systems

**DOI:** 10.3390/ijms19061788

**Published:** 2018-06-16

**Authors:** Ameya Sanjay Kasture, Thomas Hummel, Sonja Sucic, Michael Freissmuth

**Affiliations:** 1 Institute of Pharmacology and the Gaston H. Glock Research Laboratories for Exploratory Drug Development, Center of Physiology and Pharmacology, Medical University of Vienna, A-1090 Vienna, Austria; ameya.kasture@meduniwien.ac.at (A.S.K.); sonja.sucic@meduniwien.ac.at (S.S.); 2 Department of Neurobiology, University of Vienna, A-1090 Vienna, Austria; thomas.hummel@univie.ac.at

**Keywords:** *Drosophila*, dopamine, serotonin, neurodegeneration, neurotransmitter transporters, vesicular monoamine transporters

## Abstract

The brain of *Drosophila melanogaster* is comprised of some 100,000 neurons, 127 and 80 of which are dopaminergic and serotonergic, respectively. Their activity regulates behavioral functions equivalent to those in mammals, e.g., motor activity, reward and aversion, memory formation, feeding, sexual appetite, etc. Mammalian dopaminergic and serotonergic neurons are known to be heterogeneous. They differ in their projections and in their gene expression profile. A sophisticated genetic tool box is available, which allows for targeting virtually any gene with amazing precision in *Drosophila melanogaster*. Similarly, *Drosophila* genes can be replaced by their human orthologs including disease-associated alleles. Finally, genetic manipulation can be restricted to single fly neurons. This has allowed for addressing the role of individual neurons in circuits, which determine attraction and aversion, sleep and arousal, odor preference, etc. Flies harboring mutated human orthologs provide models which can be interrogated to understand the effect of the mutant protein on cell fate and neuronal connectivity. These models are also useful for proof-of-concept studies to examine the corrective action of therapeutic strategies. Finally, experiments in *Drosophila* can be readily scaled up to an extent, which allows for drug screening with reasonably high throughput.

## 1. Introduction

**Figure 1:**
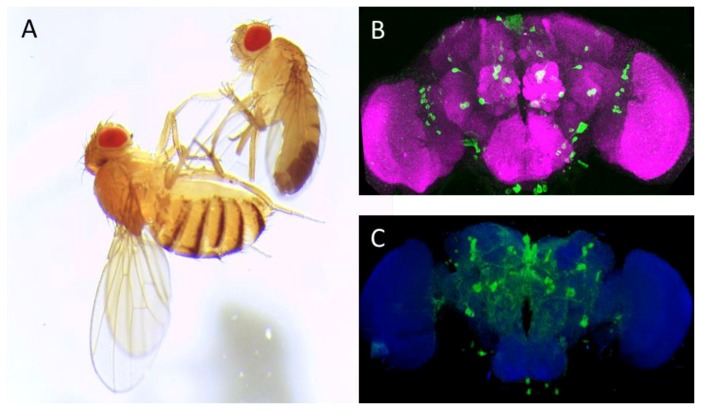
*Drosophila melanogaster* (**A**) and adult fly brain (**B**,**C**). (**A**): Image of adult female (**left**) and male (**right**) Canton-S *Drosophila melanogaster*; (**B**): Anterior view of adult fly brain carrying serotonin transporter protein trap (Bloomington Drosophila Stock Center no. 60529, Bloomington, IN, USA). The cell bodies of serotonergic neurons are labeled in **green** by expression of GFP; and (**C**): Posterior view of TH Gal4; mCD8:GFP adult fly brain: The dopaminergic neurons are delineated by their expressing the murine GFP-tagged CD8, which uniformly labels the neuronal membrane. ImageJ 3D Viewer plugin was used to generate the image. Anti-neuronal cadherin antibody (MNCD2, DSHB, University of Iowa, IA, USA, **magenta** (**B**) and **blue** (**C**)) was used to delineate the adult fly brain. Scale bar: 50 μm.

The dew-loving, black-bellied fruit fly *Drosophila melanogaster* (Figure 1A) has grown into a cherished tool used to probe biological processes over the 100 years. In his Noble Prize acceptance speech, Thomas H. Morgan, the father of *Drosophila* research, stressed the importance of *Drosophila* for genetics but humbly questioned the impact of fly biology on human physiology and pathophysiology [1]. The progress, which has been made in the past 80+ years is striking: It is clear that many fundamental biological processes are conserved between people and *Drosophila*. Along with other model organisms, such as mouse or zebrafish, *Drosophila* has greatly enriched our understanding of the human biology and medicine. Owing to its very short life cycle, the availability of rich and sophisticated genetic tools, the ease of maintenance, and more (or most) importantly cost-effectiveness, *Drosophila* embodies an “ideal” model organism.

The *Drosophila* genome comprises around 14,000 genes. These are spread over four chromosomes. Importantly, approximately 75% of all disease-related human genes have orthologues in *Drosophila* [2]. The life cycle of *Drosophila melanogaster* consists of four stages: Egg, larva, pupa, and fly. The duration of the life cycle is temperature-dependent and it is completed in about ten days when the flies are maintained at 25 °C. *Drosophila* lends itself to large-scale genetic screens [3,4,5], cell specific transgene expression [6,7,8], and precise genome editing (reviewed in [9,10]). The availability of this large genetic tool box makes *Drosophila* an ideal model organism to study conserved biological processes. Unbiased genetic screens in the flies led to the discovery of many genes, including those encoding potassium and transient receptor potential channel and clock genes. In such forward screens, random mutations are generated in the flies using chemical agents such as ethyl methyl sulfonate [11], X-ray radiation [12], or genetic means such as transposon-mediated mutagenesis [9]. The resulting mutations-carrying flies are subsequently screened for pre-defined behavioral phenotypes. The availability of single nucleotide polymorphism maps [13] and whole-genome sequencing [14] made reverse screens possible, such that the function of predefined genes can be studied. Various genetic tools, including transposable P elements [9], homologous recombination [15], and RNA mediated genome editing tools such as RNA interference [16] and CRISPR/Cas system [17] can be used for precise genome editing. The phenotype of the mutant flies can then be studied. The GAL4/UAS system, a binary ectopic expression system, can also be used to express a transgene of interest (a rescue cDNA construct or a disease relevant cDNA construct) in tissue- or cell-specific manner [7].

Remarkably, many neurotransmitters are common to *Drosophila* and mammals; i.e., dopamine, serotonin, histamine, GABA, glutamate, and acetylcholine. Invertebrates lack the dopamine β-hydroxylase, and phenylethanolamine-*N*-methyl-transferase, which are involved in the synthesis of epinephrine and norepinephrine, and instead convert tyrosine into tyramine and octopamine. The current review will focus on the monoamine neurotransmitters dopamine and serotonin and capture the current understanding of these neurotransmitter systems in flies.

## 2. The Dopaminergic System

**Figure 2:**
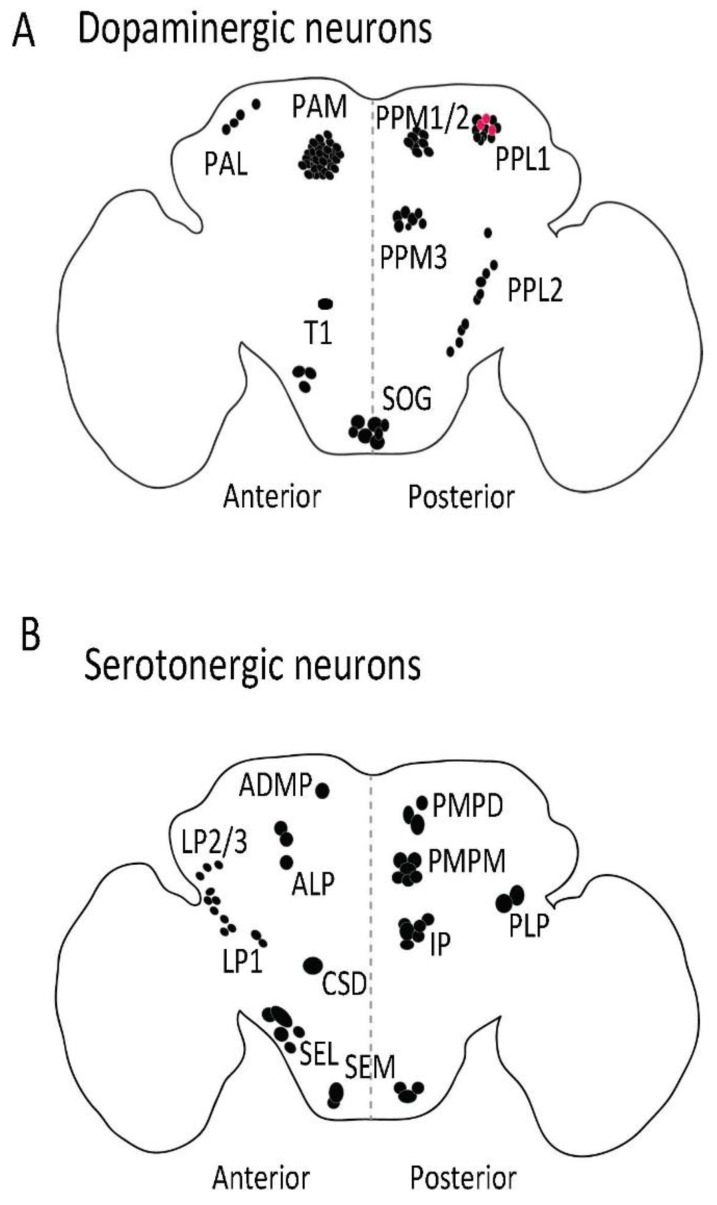
Distribution of dopaminergic (**A**) and serotonergic (**B**) neurons in adult fly brain. The dopaminergic and serotonergic neurons are distributed in various clusters in flies. Anti-TH antibody (Abcam, 128249, Cambridge, UK) was used to label dopaminergic neurons and serotonin transporter protein trap (bloomington stock no. 60529) was used to visualize serotonergic cell bodies in adult fly brain. PAM: Protocerebral anterior medial; PAL: Protocerebral anterior lateral; PPL: Posterior protocerebrum lateral; PPM: Protocerebral posterior medial; T1: Thoracic 1; SOG: Subesophagal ganglion; PLP: Posterior lateral protocerebrum; ADMP: Anterior dorsomedial protocerebrum; ALP: Anterior lateral protocerebrum; LP: Lateral protocerebrum; PMPM: Posterior medial posterior medial; SEL: Subesophageal lateral; CSD: Contralaterally projecting, serotonin-immunoreactive, deutocerebral; SEM: Medial subesophageal ganglion; PMPD: Posterior medial protocerebrum, dorsal; IP: Inferior medial protocerebral. **Red** dot: PPL1 dopaminergic neurones (**A**) that also produce serotonin. **Black** **dot**: Dopaminergic (A) and serotonergic (B) cell bodies.

The brain of *Drosophila* has around 127 bona fide dopaminergic neurons. These are spread over eight clusters per hemisphere comprised of 4 to 13 individual neurons (Figure 1C and Figure 2A) [18]; in addition, there are up to four areas with single dopaminergic neurons (in PPD, PPL3, PPL4, and PPL5); the statistical uncertainty results in the counterintuitive odd sum of 127 [18]. The dopaminergic system modulates sleep, arousal, light perception, circadian entrainment, courtship, feeding, learning, aversive conditioning, aggression, and social spacing in flies ([19,20,21,22,23,24,25,26,27], listed in Table 1). *Drosophila* express two D1-like dopamine receptors: Dop1R1 and Dop1R2, which stimulate adenylyl cyclase via the Gs subunit, and a D2-like dopamine receptor, referred to as D2R, which is Gi-coupled [28]. Flies also express one non-canonical receptor known as DopEcR, which shows appreciable affinity for both dopamine and 20-hydroxy-ecdysone, and activates different downstream signaling contingent upon the ligand in question [29]. Similar to mammals, in flies, tyrosine conversion to l-3,4-dihydroxyphenylalanine (l-dopa) is catalyzed by tyrosine hydroxylase (TH). TH is encoded by the *pale* gene; the genetic deficiency in TH is embryonically lethal [30]. The TH transcripts are alternatively spliced in a tissue-specific manner and different mRNA isoforms are expressed in the hypoderm and the central nervous system (CNS). Since flies lack melanocytes, they use dopamine synthesized in the epidermal cells to produce melatonin in the cuticle. Furthermore, dopamine metabolites *N*-β-alanyl dopamine and *N*-acetyl dopamine are involved in the hardening of the cuticle. Interestingly, TH-null lethality can be rescued by restoring the hypoderm-specific, but not by CNS-specific expression of TH [23].

TH-deficient and hence dopamine-deficient flies exhibit hypoactivity, extended sleep time, reduced arousal, lack of preference to sucrose, impaired olfactory aversive learning, and locomotor deficits which tend to worsens with age. The phenotypes shown by TH-deficient flies also remain unchanged following the treatment with a TH inhibitor, 3-iodotyrosine [23,31]. Feeding TH-deficient flies with l-dopa remedies the hypoactivity and restores sugar preference and aversive learning. These observations have two implications: (i) dopamine deficiency does not per se result in developmental defects; (ii) dopaminergic circuits remain unaffected in the TH-deficient flies. TH-deficient mice similarly show hypoactivity and reduced feeding behavior, which can be remedied by l-dopa treatment. These observations and many additional findings argue for the prospect that the conservation of the dopaminergic system in *Drosophila* and mammals allows for extrapolation from flies to men and vice versa, which when judiciously applied supports hypothesis-driven research. Overexpression of TH significantly increases male-male courtship behavior in flies [32]. This is reminiscent of hypersexuality and paraphilia, which can be induced in people treated with l-dopa and dopaminergic agonists.

During the second stage of dopamine synthesis, dopa decarboxylase (DDC) catalyzes the conversion of l-dopa to dopamine. In fact, dopa decarboxylase is aromatic amino acid decarboxylase and thus also involved in the synthesis of serotonin by decarboxylating 5-hydroxytryptophan (see below). Inhibitors of dopa decarboxylase have been developed for the treatment of people suffering from Parkinson’s disease: Carbidopa and Benserazide are useful because they do not cross the blood-brain barrier and thus limit the peripheral conversion of l-dopa. If TH-deficient flies are fed on l-dopa in combination with carbidopa, brain dopamine levels increase [31]. This observation implies that the *Drosophila* blood-brain barrier acts in a similar manner as the mammalian blood-brain barrier: It precludes permeation of exogenous compounds (i.e., carbidopa) and endogenous neurotransmitters (i.e., dopamine, which is present in the hemolymph of the flies) into the brain.

In mammals, the vesicular monoamine transporters (VMAT1/SLC18A1; VMAT2/SLC18A2) store newly synthesized dopamine, serotonin, and adrenaline/noradrenaline in synaptic vesicles and large dense core vesicles for their ensuing synaptic and extra-synaptic release [33]. *Drosophila* has only one vesicular monoamine transporter (*dVMAT*) gene, which produces two splice variants with distinct C-termini, dVMAT-A and dVMAT-B [34]: dVMAT-A is expressed in dopaminergic, octopaminergic, and serotonergic neurons [34]; in contrast, dVMAT-B is present in a small population of optic glia and plays role in histamine homeostasis [35]. There is no mammalian equivalent to glial expression of a VMAT isoform. The role of neuronal dVMAT-A is understood in considerable detail; in fact, the elaborate toolbox of *Drosophila* research allows for neuron-specific re-expression of dVMAT in a dVMAT-null background. Hence, it is possible to interrogate dVMAT-deficient flies to understand the redundancy and complementary function of the three monoamines [36]: Dopamine is, for instance, important in male courtship but it is redundant, because it can be replaced by octopaminergic signaling. The circadian rhythm, on the other hand, depends on the cooperation of at least two monoamine neurotransmitters. The experiments also revealed that vesicular release of both dopamine and serotonin, but not of octopamine, is dispensable during larval development and adult survival.

In neurons, membrane depolarization triggers Ca^2^⁺ influx into the intracellular compartment of the presynaptic membrane. Subsequently, synaptic vesicles filled with dopamine fuse with the membrane in order to release dopamine into the synaptic cleft. Upon signal transmission, dopamine is rapidly taken up by the dopamine transporter (DAT), which works in relay with VMATs to replenish the vesicular dopamine storage. DAT is a target of important therapeutic agents such as methylphenidate, which is prescribed for narcolepsy and attention deficit hyperactivity disorder, as well as substances of abuse, such as cocaine and amphetamines [37]. *Drosophila* DAT (dDAT) and human DAT (hDAT) exhibit similar substrate specificity. However, the pharmacological profile of dDAT resembles that of the human norepinephrine transporter [38]; dDAT also has a lower affinity for cocaine than hDAT, but application of cocaine blocks the reuptake of endogenous dopamine in larvae and adult flies [39,40]. Accordingly, *Drosophila* can be used to study the action of addictive drugs. Amphetamine and its congeners switch monoamine transporters from a forward transport mode to a substrate exchange mode [37]. This is contingent on transporter phosphorylationby CaM-kinase II [41,42] and requires the N-terminus [43,44]. Several of these findings can be recapitulated by examining the action of amphetamine in flies: Amphetamine induces hyperactivity in flies, which is mediated by dDAT-dependent dopamine efflux and requires phosphorylation by CaM-kinase II [41,42,45,46,47]. Most importantly, the experiments pointed to an important role of flotillin-1 in supporting the action of amphetamine and thus identified a component, which had hitherto not been appreciated [41].

DAT-null flies are viable and hyperactive. Normal sleep duration in flies is some 16 h/day. In contrast, DAT-null flies sleep only 5–6 h/day [47]. This loss of function phenotype has become a useful tool addressing some important questions, which are relevant to human diseases: The substitution of M for T in codon 356 was identified as one the mutations of hDAT, which occur in patients with autism [48]. When expressed heterologously in cultured cells, hDAT-T356M gave rise to sustained dopamine efflux [49]. Transgenic flies expressing hDAT-T356M (in the DAT-null background) display the hyperactivity behavior reminiscent of that shown by DAT-null flies [49]. Taken together, these finding indicate that the hDAT-T356M mutation disrupts the forward transport mode and thus precludes dopamine uptake, which accounts for the hyperactivity of flies. It is also plausible to assume that in a heterozygote person (harboring alleles encoding hDAT-T356M and wild type DAT) the abnormal properties of hDAT-T356M drives an abnormal dopaminergic tone, which gives rise to an autism spectrum disorder.

The genetic toolbox, which is available for of *Drosophila*, does not only allow for analyzing disease-causing mutations, it can also be applied to search for therapeutic strategies. As a proof of concept, we examined the dDAT-G108Q mutation, which was created flies by random mutagenesis and which produced a reduced sleep phenotype [46]. The heterologously expressed mutant protein is trapped in the endoplasmic reticulum (ER), as a consequence dopamine uptake is abolished [45]. We tested two pharmacological approaches to restore the cell surface expression of this folding-deficient mutant: (i) relaxing the protein folding quality-control mechanisms in the ER by inhibiting HSP70 using pifithrin-µ [45,50]; (ii) pharmacochaperoning with noribogaine, which binds to and stabilizes monoamine transporters in the inward-facing state [51] The rationale of these two approaches is based on studies with SERT folding mutants [50,52,53,54,55]. In fact, in transfected cells, both noribogaine and pifithrin-µ restored surface expression to and dopamine uptake by dDAT-G108Q and its human equivalent hDAT-G140Q [45]. Most importantly, administration of noribogaine or of pifithrin-µ and of their combination also corrected the deficiency in flies: The mutant protein was delivered to the axonal territory and sleep duration increased to the normal range [45]. Misfolded DAT variants occur in people: There are 15 point mutations in human DAT, which all give rise to misfolding of the protein. The clinical picture of the dopamine transporter deficiency syndrome (DTDS) is a movement disorder of infancy and childhood with dystonia and parkinsonism [56,57,58,59]. DTDS is not the only protein folding disease in the SLC6 transporter family [60], but it is currently the disease which can be most readily studied in flies [61,62,63]. Two DTDS-causing mutants (hDAT-V158F and hDAT-G327R) were amenable to functional rescue by noribogaine and pifithrin-µ in living flies: Pharmacochaperoning restored axonal delivery of the otherwise ER-retained mutant protein and corrected the sleepless phenotype [61,62,63]. Another research group reported that some of the mutants are also responsive to pharmacological rescue by bupropion [64], although this finding has not been confirmed in behavioral assays in flies. Monoamine transporters have a rich pharmacology comprising atypical inhibitors and partial substrates, i.e., compounds, which trap the transporters at different stages of their conformational cycle [37]. Partial substrates of monoamine transporters can also act as pharmacochaperones but their effectiveness may be limited by their high affinity [65]. Experiments in transgenic flies expressing DTDS variants thus provide a litmus test to interrogate potential new pharmacochaperones for properties, which make them useful in vivo.

In the mammalian brain, the vast majority of dopaminergic neurons reside in three anatomically distinct, but adjacent regions in the midbrain, i.e., the substantian nigra pars compacta (A9), the ventral tegmental area (VTA/A10), and the retrorubral area (A8). Neurons from these three individual areas differ in their projections. The A9 and A10 region has been further subdivided into five and seven subgroups, respectively, based on anatomical/cytoarchitectonic criteria. More recently, gene expression profiling has allowed for classifying individual neurons based on their complement of transcripts [66]. This exercise shows a large heterogeneity of dopaminergic neurons with respect to the expression of ion channels, receptors, transcription factors, and secretory peptides, allowing for the classification of at least six distinct subpopulations [66]. The functional heterogeneity of dopaminergic neurons can be readily addressed by the available genetic toolbox in *Drosophila*. This can be exemplified by examining the handling of aversive and attractive stimuli and the regulation of the sleep-wake cycle: 

(i) In mammals, dopaminergic signaling—in particular from the ventral tegmental area—marks salient features of sensory input and is hence associated with pleasure, reward, and aversion [67,68]. Neurons from the ventral tegmental area also participate in a circuit, which controls the mammalian sleep–wake cycle. In flies, these pathways have been dissected to yield a detailed understanding of input/output-relation, decision-making, and memory formation [18,21,22,69,70,71,72,73,74,75]: Dopaminergic input impinges on the mushroom body output neurons: PPL1 dopaminergic neurons are involved in aversive olfactory reinforcement [76,77]. PAM dopaminergic neurons promote reward reinforcement [22,71,72,78]. Both dopaminergic PAM and PPL1 neurons are subject to feedback control by mushroom body output neurons. This allows for a re-evaluation of an olfactory memory and thus for situation-dependent re-consolidation or extinction [75]. The signaling pathways underlying memory formation and extinction have also been addressed in two dopaminergic neurons residing in PPL1 (MP1 and MV1), which show synchronized ongoing activity before and after learning. Memory acquisition and forgetting depend on Dop1R1 (or dDA1) and Dop1R2 (or DAMB), respectively [70]. In addition to coupling with Gs, Dop1R2 also signals via Gq, which mediates the forgetting of memory [79].

(ii) Sleep regulation in flies relies on the fan-shaped body [62,80,81,82,83]. Dopamine promotes arousal; the neurons involved are somewhat controversial as are the postsynaptic receptors and signaling mechanisms: A single pair of dopaminergic neurons from PPL1 [81], PPM3 [83], or both, suffices to drive the arousal reaction. It was originally proposed that the dopamine engaged Dop1R1 and a Gs/cAMP-dependent pathway [81,83]. More recently, a (non-canonical, pertussis-toxin-sensitive) G_i_/G_o_-mediated signaling via Dop1R2 was shown to elicit two distinct effects on the postsynaptic neurons of the fan-shaped body, which operate on different time scales: An instant—but transient—hyperpolarization, which was due to stimulation of one or several voltage-activated K⁺ channels, and an off-state, which relies on the exocytosis of an internal, vesicular store of a two-pore-domain K^+^ channel; this K_2P_ channel was termed *Sandman* [80]. Insertion of *Sandman* produces a large leak current, which silences the fan-shaped body neurons and thus produces a long-lasting state of wakefulness [80].

It is evident that these insights are informative for addressing the question, how the human brain generates sleep and marks saliency for writing and erasure of memory. It is also worth noting that human diseases can be modeled in *Drosophila* by either targeting the gene of interest or by overexpressing disease causing human orthologs: The list includes Parkinson’s disease, Alzheimer’s disease, and spinocerebellar ataxia 2 and 3 [84,85,86,87]. For example, overexpression in flies of human *α-synuclein* (the first gene discovered to be linked to familial Parkinson disease [88]) results in age-dependent loss of dopaminergic neurons and impaired locomotor activities [89].

## 3. The Serotonergic System

*Drosophila* has approximately 80 serotonergic neurons, spread over various clusters (Figure 1B and Figure 2B) [90]. The serotonergic system regulates sleep, place memory, circadian rhythm, feeding, aggression, nociception, and long-term memory formation ([90,91,92,93,94,95], listed in Table 1). An additional pair of neurons was recently reported, the location of which coincides with the PPL1 dopaminergic cluster and expresses SERT [96]. These neurons may contain both dopamine and serotonin [97] (Figure 2B, represented as red spheres). *Drosophila* expresses five G protein-coupled serotonin receptors, G_i_/G_o_-coupled 5HT-R1A and -1B, the Gq-coupled 5HT-R2A and -2B, and the Gs-coupled 5HT-R7 [98,99,100,101,102].

Serotonin is synthesized in serotonergic neurons in a two-step process. Firstly, in the rate limiting step, tryptophan hydroxylase (TRH) converts l-tryptophan to 5-hydroxytryptophan, which, in the second step, is cleaved by the namesake action of DDC/aromatic amino acid decarboxylase to yield serotonin. TRH is responsible for producing neuronal serotonin, while tryptophan phenylalanine hydroxylase (TPH) encoded by *Henna* hydroxylates tryptophan as well as phenylalanine to generate peripheral serotonin and tyrosine [103]. Interestingly, TPH is required for early development, implying that both serotonin and dopamine are required in non-neuronal cells during development itself [23,104,105]. TRH-null flies are viable and fertile with diminished locomotion and feeding behavior [105]. Overexpression of TRH in serotonergic neurons results in increased cytoplasmic serotonin levels, leading to spheroid formation, i.e., large aberrant swelling in neurites [106]. Interestingly, this TRH overexpression-mediated spheroid formation is specific to serotonergic neurons: TRH overexpression in dopaminergic neurons, which also express DDC and hence produce serotonin, does not result in spheroid formation [106]. Mutation in DDC causes extensive branching of serotonergic axonal arborizations in larvae [107]. Deletion of VMAT (see above) does not interfere with viability or fertility; this implies that serotonin release is dispensable for viability and fertility [36]. However, serotonin is required for the late startle response and for the circadian rhythm [36]. The circadian rhythm can be rescued by restoring VMAT expression in at least two of the three monoaminergic systems indicating cooperation between and redundancy in serotoninergic, dopaminergic, and octopaminergic neurons. In contrast, while there is a cooperation of monoaminergic neurons in the startle response, it is not functionally redundant such that the late startle response depends on serotonin [36]. Excess of cytoplasmic serotonin can alter the morphology of serotonergic neurons [106]. The released serotonin is retrieved by SERT located presynaptically and extra-synaptically. dSERT and hSERT display similar but not identical pharmacological properties [108]. For instance, selective serotonin reuptake inhibitors (SSRI) such as paroxetine, fluoxetine, and citalopram bind to dSERT with lower affinity than to hSERT [108]. Nevertheless, serotonin and cocaine exhibit similar affinities for dSERT and hSERT [108].

In *Drosophila* the RNA encoding *dSERT* is expressed late in embryonic development (stage 15), just before serotonin is produced (stage 17) [108]. A recent study characterized a SERT-hypomorph mutant *Drosophila*, which expresses SERT at reduced levels [109]: Flies harboring the SERT mutant display increased basal locomotion and reduced centrophobism, i.e., anxiety-like behavior [109]. This may appear counterintuitive, because reduction in neuronal serotonin is associated with decreased locomotion [105], but it highlights the doubled-edged nature of transporter deficiency/inhibition: The plasmalemmal monoamine transporters are required for both termination of synaptic signaling by removing the neurotransmitter and for replenishing the vesicular pool by operating in relay with VMAT. The symptoms of Parkinsonism, which are seen in the human dopamine transporter deficiency syndrome (see above), highlight the importance of the dopamine transporter in maintaining the vesicular dopamine pool. It is evident that the genetic toolbox of *Drosophila* allows for examining the dichotomy of synaptic clearance and storage pool maintenance.

The serotonergic system of *Drosophila* has not been explored to the same extent as the dopaminergic system. This can be accounted for by the absence of distinct phenotypic consequences resulting from depletion of serotonin in flies. However, a number of recent studies have started dissecting the role of individual serotonergic circuits and their role/s in various physiological processes [110,111,112,113]. Interestingly, these analyses revealed that activation of individual subsets of serotonergic neurons results in behavioral consequences different from those elicited by global serotonergic stimulation [110,111,112]. A subset of serotonergic neurons encodes hunger; their selective activation promotes feeding even in the sated state [110]. In contrast, global activation of serotonergic neurons elicits the opposite response [110]. Flies do not differ from mammals in this respect: Global serotonergic stimuli (e.g., the serotonin releaser MDMA/methylenedioxymethamphetamine/ecstasy) also cut the appetite in people and rodents. Similarly, bidirectional changes are also seen in odor perception: Attraction to ethanol is suppressed by stimulation of four distinct serotoninergic neurons (in IP and LP1, shown in Figure 2B); two distinct neurons (CSD, see Figure 2B) counteract their action and thus restore suppressed ethanol attraction, when activated [111].

Dysregulation of the serotonergic system in people can result in mood disorders, anxiety, and bulimia and anorexia nervosa. Flies are capable of performing complex behavioral tasks. A recent study used a chronic stress paradigm to induce a depression-like state in flies [113]. The researchers subjected flies to repetitive episodes of uncontrollable mechanical stress for three to four days and assessed their climbing motivation. The stressed flies performed less climbing attempts compared to the unstressed flies. The stressed flies showed reduced activity in voluntary behaviors, including locomotion and initiation of courtship, while maintaining non-motivational behaviors, e.g., the escape response. The climbing behavioral deficit was restored upon treatment with a selective serotonin reuptake inhibitor fluoxetine, as well as by sugar treatment [113]. The combination of genetic and pharmacological tools allowed for assigning this modulation of climbing motivation to serotonergic neurons projecting to α and γ lobes of the mushroom body, a brain region equivalent to human hippocampus [113]. Arguably, this behavioral read-out can be employed for drug screening in flies expressing human orthologs and clinically relevant variants thereof.

## 4. Cross-Talk between the Dopaminergic and Serotonergic Systems

Evidence of interactions between dopaminergic and serotonergic system has repeatedly been reported in mammalian and invertebrate systems [97,114,115,116,117]. In flies, most neuropils are innervated by both dopaminergic and serotonergic neurons with a few exceptions. Interestingly, a small population of fly neurons shows a positive immunoreactivity for both dopamine and serotonin [97]. A general consensus in both mammalian and invertebrate field is that at the system level, serotonin acts as an inhibitor of behavior [118,119], whereas dopamine is primarily involved in arousal [120,121]. For instance, dopamine-deficient flies show a reduced locomotion phenotype [23,31], which can be mimicked via the activation of serotonergic neurons [112]. A recent study, performed in flies, revealed a role of a serotonin-dopamine axis in long-term memory formation [95]: A pair of newly identified bilateral serotonergic projection neurons control the activity of MP1 dopaminergic neurons, which are involved in long-term memory formation. Inhibition of Dunce phosphodiesterase, which restricts the cAMP levels at the presynaptic region and hence the cAMP/PKA signaling [122], in serotonergic neurons results in serotonin release which activates 5HT-2A receptors in MP1 dopaminergic neurons, subsequently resulting in long-term memory formation at the mushroom body [95]. DDC mutant flies, which lack neuronal dopamine and serotonin, have an extended serotonergic arborization in the larval stage: This was, in part, suppressed by dopamine feeding [107]. In neonatal rodents, 6-hydroxydopamine lesioning is commonly used in combination with a norepinephrine reuptake inhibitor to selectively target dopaminergic neurons [123]. The resulting selective loss of dopaminergic neurons affects the serotonergic innervations in a region-specific manner [124,125]. In addition, the extensive serotonergic arbor plasticity is not limited to developing brains, since a selective loss of dopaminergic neurons in the adult mouse brain also leads to extensive sprouting of serotonergic processes [126]. Aberrant serotonergic signaling—resulting from a change in dopaminergic input and vice versa—is thought to contribute to the evolution of several disorders including depression, schizophrenia, and Parkinson’s disease (reviewed in [127]). Consistent with findings in the dopamine-deficient mammalian brain, the serotonergic neurons sprout in dopamine-deficient flies; this results in increased density of projections to territories in the mushroom body, which are sparsely innervated by serotoninergic neurons in wild type flies [97]. The competitive interaction between dopaminergic and serotoninergic projections is further substantiated by the observation that the serotoninergic projections are rarefied in the mushroom body of wild type flies, after these have been fed l-dopa for 10 days [97]. These findings suggest that the serotonin-dopamine interactions are conserved across species and over large evolutionary distances. It is therefore justified to consider *Drosophila* a suitable model for studying aberrant dopamine and serotonergic signaling.

Excitatory neurotransmission in the brain relies on glutamate. Dopamine and serotonin also impinge on glutamatergic synapses and this cross-talk is thought to be important in several psychiatric disorders, e.g., schizophrenia, autism, bipolar depression [128,129]. It appears counterintuitive to use the simple brain *Drosophila* as a model organism for studying complex disorders of higher brain function in man. In addition, protostome invertebrate phyla express glutamate-gated chloride channels. Thus, in contrast to mammals, glutamate can act in both, excitatory and inhibitory manner in *Drosophila* [130,131,132]. Nevertheless, it is possible to capitalize on *Drosophila* to analyze components of regulatory circuits: This is epitomized by experiments designed to understand the impact of dysbindin/dystrobrevin binding protein 1, the product of a schizophrenia susceptibility gene in people. In both mouse [133] and flies [134], reduced expression of dysbindin results in a hyperdopaminergic and hypoglutamatergic phenotype. The crucial insight from *Drosophila* was the finding that this alteration resulted from the action of dysbindin in to different cellular compartments, i.e., neuronal and glial for the hypoglutamatergic and heyperdopaminergic signaling, respectively [134].

## 5. Conclusions

A collection of sophisticated genetic tools allows for spatial and temporal control over gene expression in *Drosophila melanogaster*. In addition, flies can perform complex tasks by relying on some 100,000 neurons in their brain. It is therefore possible to map neural circuits supporting individual aspects of behavior and the required decision making and memory formation with amazing precision. In comparison to mammals (e.g., mice, which have around 30–35,000 dopaminergic [135] and 26,000 serotonergic neurons [136]), flies have a small number of dopaminergic and serotonergic neurons. These are grouped into clusters and their widespread neurites reveal a substantial degree of overlap. *Drosophila* provides a unique opportunity to understand the role of serotonergic and dopaminergic transmission in a reductionist approach. Firstly, large scale screens can be performed in a cost- and time-efficient manner. Secondly, the capacity to introduce human orthologs allows for studying the encoded proteins including disease-associated variants in vivo. Finally, *Drosophila* is a very elegant animal model, not only for understanding biological processes, but also for performing drug screening. It is self-evident that every model has its limitations: A striking difference between mammalian and invertebrate neurons is their distinct morphology: Fly neurons are unipolar; a single neurite projects from the cell body and gives rise either to axonal and dendritic arborization or to an axonal shaft with multiple projections [137]. In mammals, neurons are predominantly bi- or multipolar, i.e., dendrites and axons originate separately from the cell body. In addition, and by definition, a reductionist approach aims at eliminating complexity. The number of possible connections rises exponentially as the number of neurons increases. Thus, there are inherent limits to what the *Drosophila* brain can teach us about the human brain and its complex disorders, e.g., schizophrenia, depression and autism.

## Figures and Tables

**Table 1:** Dopaminergic and serotonergic neurons involved in various biological functions.

Neuronal Cluster	Function	Ref.
Dopaminergic neurons		
PAM	Sugar reward, reward signaling, long-term and short-term memory formation, aversive memory formation, negative geotaxis, promote wakefulness, foraging behavior, promote oviposition preference	[22,71,77,138,139,140,141]
PAL	Involved in mating drive	[142,143,144]
PPL1	Aversive memory formation, sugar reward and nutrient value, negative geotaxis, modulate sleep, Inhibit oviposition preference	[1,3,4,5,7,11,12,145]
PPM2	Protein consumption preference	[146]
PPM3	Aggression phenotype Modulate sleep, promote oviposition preference	[7,14,15,16]
T1	Aggression phenotype	[21]
SOG	Proboscis extension in response to satiety state	[147]
Serotonergic neurons		
PLP	Aggression	[148]
PMPM	Modulate anesthesia resistant memory and sleep	[149,150]
CSD	Modulate ethanol perception	[111]
SEL	Long term memory formation	[95]

Neuronal circuitry has been functionally linked to a number of dopaminergic and serotonergic neurons belonging to different clusters. Similar to mammals, flies carry monoaminergic neurons organized into clusters and the neurons vary in their anatomy and postsynaptic targets. PAM: Protocerebral anterior medial; PAL: Protocerebral anterior lateral; PPL1: Posterior protocerebrum lateral; PPM: Protocerebral posterior medial; SOG: Subesophagal ganglion; PLP: Posterior lateral protocerebrum; PMPM: Posterior medial posterior medial; SEL: Subesophageal lateral; CSD: Contralaterally projecting, serotonin-immunoreactive, deutocerebral.
